# Accidental hypothermia in Poland – estimation of prevalence, diagnostic methods and treatment

**DOI:** 10.1186/s13049-014-0086-7

**Published:** 2015-02-06

**Authors:** Sylweriusz Kosiński, Tomasz Darocha, Robert Gałązkowski, Rafał Drwiła

**Affiliations:** Department of Anesthesiology and Intensive Care, Pulmonary Hospital, Zakopane, Poland; Tatra Mountain Rescue Service, Zakopane, Poland; Department of Anesthesiology and Intensive Care, John Paul II Hospital, Collegium Medicum, Jagiellonian University, Cracow, Poland; Department of Cardiac Surgery, John Paul II Hospital, Collegium Medicum, Jagiellonian University, Cracow, Poland; Department of Emergency Medical Services, Medical University of Warsaw, Polish Medical Air Rescue, Warsaw, Poland

**Keywords:** Accidental hypothermia, Prevalence, Rewarming, Thermometers, Body temperature

## Abstract

**Background:**

The incidence of hypothermia is difficult to evaluate, and the data concerning the morbidity and mortality rates do not seem to fully represent the problem. The aim of the study was to estimate the actual prevalence of accidental hypothermia in Poland, as well as the methods of diagnosis and management procedures used in emergency rooms (ERs).

**Methods:**

A specially designed questionnaire, consisting of 14 questions, was mailed to all the 223 emergency rooms (ER) in Poland. The questions concerned the incidence, methods of diagnosis and risk factors, as well as the rewarming methods used and available measurement instruments.

**Results:**

The analysis involved data from 42 ERs providing emergency healthcare for the population of 5 305 000. The prevalence of accidental hypothermia may have been 5.05 cases per 100.000 residents per year. Among the 268 cases listed 25% were diagnosed with codes T68, T69 or X31, and in 75% hypothermia was neither included nor assigned a code in the final diagnosis. The most frequent cause of hypothermia was exposure to cold air alongside ethanol abuse (68%). Peripheral temperature was measured in 57%, core temperature measurement was taken in 29% of the patients. Peripheral temperature was measured most often at the axilla, while core temperature measurement was predominantly taken rectally. Mild hypothermia was diagnosed in 75.5% of the patients, moderate (32-28°C) in 16.5%, while severe hypothermia (less than 28°C) in 8% of the cases. Cardiopulmonary resuscitation was carried out in 7.5% of the patients. The treatment involved mainly warmed intravenous fluids (83.5%) and active external rewarming measures (70%). In no case was extracorporeal rewarming put to use.

**Conclusions:**

The actual incidence of accidental hypothermia in Polish emergency departments may exceed up to four times the official data. Core temperature is taken only in one third of the patients, the treatment of hypothermic patients is rarely conducted in intensive care wards and extracorporeal rewarming techniques are not used. It may be expected that personnel education and the development of management procedures will brighten the prognosis and increase the survival rate in accidental hypothermia.

## Introduction

The incidence of accidental hypothermia is difficult to estimate. To a great extent the difficulties result from a lack of unified guidelines for temperature measurement and hypothermia classification, as well as scarce diagnostic algorithms. According to the official data of the Central Statistical Office for the period of 2009–2012, hypothermia was the primary cause of death in 1836 residents in Poland and in 489 (26.63%) cases death was pronounced in the hospital.

Diagnosis of low temperature-related conditions is rarely considered, particularly in regions, where the risk of low temperature exposure is low. Thus, the data concerning the morbidity and mortality rates of hypothermia seem to under-represent the problem. Such divergence may particularly concern the cases where hypothermia emerges subsequently to diseases and conditions impairing thermoregulation.

The aim of the study was to estimate the actual prevalence of accidental hypothermia in Poland, as well as to identify methods of diagnosis and management procedures in emergency rooms (ERs). We decided to collect data directly from ERs with a specially designed questionnaire. It was expected that the information collected would constitute the basis for analysing the current status of the problem in Poland, as well as the reasonableness of establishing facilities designated specifically for the treatment of severe accidental hypothermia.

## Methods

According to the Regional Ethical Review Board, the study did not require review. Research was carried out in May 2012. The questionnaire was supplied to the management teams of all the 223 ERs in the country. In the Polish Emergency Medical System (EMS) each ward functions to the same level with regards to qualifications, equipment and human resources and independently on level of the hospital. The collection form included 14 questions on the prevalence, diagnostic methods and hypothermia risk factors, as well as the methods applied for rewarming. The subjects were identified in hospital databases by means of the International Classification of Diseases, version 10, using the codes T68, T69 or X31. Patient records were reviewed by medical personnel and the data required were entered into the collection form provided by the authors. The majority of the questions demanded the provision of figures, and one question was focused on the equipment of ERs, including measurement instruments. We assumed that core temperature is taken by means of a thermistor probe in the oesophagus, rectum, urinary bladder, or on the tympanic membrane. Three questions were accompanied by explanations. Emphasis was put on the aetiology of hypothermia – the data provided could not include post-traumatic hypothermia. A request was also included to review the ED’s database in search of patients in whom hypothermia developed concurrently with other diseases and was not included or described with a relevant code in the final diagnosis. Each questionnaire required answers from medical personnel only (managing physician or nurse). The filled out data collection forms were reviewed and categorised by the authors.

Descriptive statistics were provided as mean values and a standard deviation or percentage. The chi-square test was used in order to define the uniformity of the distribution of answers. Assumption was made that the values expected for each category of the variables analysed were equal. The same significance level of 0.01 was approved for all the calculations. The official data of the Main Statistical Office were used as a base for complementary analyses of the quality of life as a risk factor for hypothermia. Statistical analyses were performed with SPSS 17.0.0.

## Results

From each of the sixteen provinces in Poland two to seven questionnaires were sent back. In total 50 collection forms were thus acquired, however eight were not completed. Therefore, data from 42 ERs were submitted for the analysis. The analysed ERs provided healthcare to a combined population of 5 305 000 people. In total, 268 cases of hypothermia were reported in the year 2011. The diagnosis of T68, T69 or X31 was entered into the documentation of 67 patients (25%). In 201 (75%) cases, hypothermia developed concurrently with other diseases and was not included or described with a relevant code in the final diagnosis. Hypothermia was not diagnosed at all in 9 ERs. Therefore, the prevalence of accidental hypothermia may have been 5.05 cases per 100 000 residents per year in the population analysed. The causes of hypothermia are listed in Table 1. Poor social living conditions (separation, negligence and poverty) were found in 66 cases (24.8%), and 106 persons (39.8%) were homeless. The complementary analysis showed that the poverty level, with the assumed threshold of 14%, in a particular province did not affect the prevalence of hypothermia (χ^2^ = 6.89, p = 0.03). The lower urbanization level of a province (<63%) turned out to be a contributory risk factor for hypothermia (χ^2^ = 12.77; p = 0.002).

**Table 1:** **Causes of hypothermia**

***Cause***	***Number***	***%***
Cold air exposure in alcohol intoxication	182	67.9
Cold air exposure	52	19.4
Cold water immersion	17	6
Substances impairing thermoregulation	4	1.5
Other	13	4.8
**In total**	**268**	

The most commonly applied method of diagnosing hypothermia was the measurement of peripheral temperature (153 patients, 57,1%). Core temperature was taken in 78 patients (29.1%), amongst the remaining cases diagnosis was made on the basis of clinical examination (37 patients, 13.8%). The distribution of the responses was not uniform (χ^2^ = 61.00; p = 0.000). Peripheral temperature was measured most often at the axilla and core temperature in the rectum (Table 2).

**Table 2:** **Core temperature measurement**

***Site***	***Number***	***%***
Rectal	43	49,4
Oesophageal	35	40,2
Tympanic	9	10,3
**In total**	**87**	

Mild hypothermia was diagnosed in the majority of patients (202 patients, 75.4%). Moderate hypothermia (32-28°C) was found in 44 (16.4%), and severe (<28°C) in 22 patients (8.2%). The lowest recorded temperature was 25°C. The mean of the lowest temperatures reported across ERs was 29.1°C (SD = 3.2°C).

**Figure 1:**
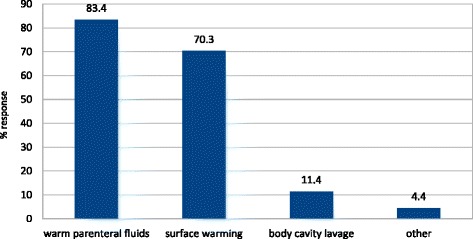
**Methods of rewarming.**

The treatment of hypothermia relied mostly on warmed intravenous fluids, yet usually various rewarming techniques were employed at the same time (Figure 1). Airway warming was instituted in a single ER in 10 cases (4.4% of the indications) and in no case was extracorporeal rewarming implemented.

About half of the patients were treated at the ER and discharged home and only 8.2% of the patients were transferred to intensive care units (Table 3). Cardiopulmonary resuscitation was carried out in 20 patients (7.5%), with the longest efforts lasting up to 7 hours. The mean longest time of resuscitation was 141.5 minutes (SD = 86.4 minutes).

**Table 3:** **The process of treatment**

***Course***	***Number***	***%***
ER and discharges	133	49,6
ER and transfer to other wards	98	36,6
ER and transfer to ICU	22	8,2
ER and transfer to other facilities	8	3
Death at ER	7	2.6
**In total**	**268**	

Measurement instrument data were obtained from 35 ERs. Infrared emission detection (IRED) thermometers were the most commonly used devices (19 ERs, 54.3%). Rectal and oesophageal probes were the dominant tool for core temperature assessment (27 departments, 77.1%). There were no urinary bladder probes, nor probes for epitympanic measurement.

## Discussion

The US National Hospital Ambulatory Medical Care Survey showed that hypothermia or other low temperature – related conditions were diagnosed in 4 persons per million inhabitants annually in ERs between 1995–2004 period [1]. The prevalence of hypothermia in European countries is 0.13-5.3 cases per 100 thousand inhabitants per year [2-5] and 6.9 per 100 thousand inhabitants per year in New Zealand [6]. In west Scotland hypothermia was diagnosed during the winter of 1993/1994 in one per 14.000 of the admitted patients. Translating these data to the whole population of the UK, hypothermia might have been a reason for 4000 admissions at ERs and for as many as 1000 deaths, whereas, national data bases in England and Wales show that hypothermia causes the death of 300 people per year [7]. Official rates may be far lower than the actual prevalence of the condition, which is also confirmed by the results of our study [1,2,6,7]. Accidental hypothermia was officially diagnosed in 1.26 per 100 000 residents in Poland, but in fact it was probably four times more frequent. The questionnaire which was used in the study was designed to expose this difference. Nevertheless, these are still estimates often based on retrospective data and clinical examination, frequently not confirmed by body temperature measurement.

The main cause of heat loss in our study was the exposure to cold air (almost 90% of the cases) and only 6% of the patients suffered from hypothermia due to cold water immersion. By comparison – this proportion was as high as 30% in the Netherlands [2]. Likewise within other studies [2,4,7,8] hypothermia was often associated with alcohol intoxication – more than 2/3 of patients were affected by ethanol. Social living conditions were also associated with the development of hypothermia. Social isolation, negligence or poverty were found in about 25% of the patients with hypothermia and nearly 40% of victims were homeless. The complementary analysis of social and economic aspects focused on two objective indicators of the quality of life. We did not find a statistically higher prevalence of hypothermia in provinces with higher levels of poverty however we succeeded in showing a relationship between worse urbanization rates and the prevalence of hypothermia. Both of these observations are surprising – since considering the high prevalence of hypothermia among the homeless and the poor, a completely opposite result was expected. One of the reasons behind this result might be the fact that perhaps we used inappropriate criteria or the number and distribution of data was not sufficient for a credible analysis, but we believe that this finding needs to be explained and further detailed studies are required.

The diagnosis of hypothermia was based on peripheral temperature measurement and a clinical examination in two thirds of the cases. Core temperature was only taken in less than one third of patients. It is worth pointing out that according to international and local recommendations, core temperature is crucial for the diagnosis of hypothermia [9-11]. Additionally, both the classification and treatment algorithms are based on the temperature of internal organs. To explain this paradox we undertook the analysis of available measurement instruments at ERs. Pursuant to effective regulations in Poland, each ER should be equipped with a device for core temperature assessment. In the majority of departments, it was declared that monitor probes are used for oesophageal or rectal measurements. None of the ERs declared using epitympanic probes, although these are considered to be optimal in terms of accuracy and low invasiveness [9,12]. Nor were there any urinary bladder probes. The usefulness of classic, hand-held and IRED thermometers for the measurement of central temperature is limited in accidental hypothermia [9,13]. Consequently, the reliable evaluation and classification of hypothermia might not have been compromised in 8 out of 35 departments (22.85%).

Another factor we have taken into consideration is the simple lack of personnel expertise in accidental hypothermia. Some of the blank questionnaires bore a note: “We do not treat hypothermia at our ER” which indicate a complete lack of knowledge in this matter. This finding is reinforced by the fact that nine out of 50 ERs did not diagnose hypothermia at all, which is unlikely. We are aware that data on hypothermia classification based on such imprecise evaluation cannot be completely reliable, but on the other hand the lack of awareness and wrong diagnostic pathways present in Polish emergency rooms are one the findings of the study. It seems, however, that problems with proper and accurate measurement may occur in other emergency medical systems too [5,11].

About half of the patients were treated at ER and discharged home. Only 8% of them were transferred to intensive care units (ICU). However, based on the experience of leading medical centres in Europe, the ICU is the optimal ward for treating patients with hypothermia [4,14-17]. Moderate or severe hypothermia (<32°C) was diagnosed in one out of four patients who were admitted to Polish ERs, which means that treatment at intensive care units could have been fully justified.

The mortality rate within the group analysed was 6.3%. It is worth highlighting however, that this data refers only to the period patients spent in ERs – and the follow-up history of the patients who were transferred to other departments remains unknown. At the University Hospital in Vienna a total 5% of 80 patients with diagnosed accidental hypothermia died at the ER, but the overall mortality rate was 34% [16]. Similar mortality rates were observed at the University Hospital in Amsterdam [17].

The most commonly used method of treatment was by means of warmed intravenous infusions and about two thirds of the patients were rewarmed by means of forced air warming or carbon-fiber blankets. In general, several rewarming options using a variety of means to deliver heat were reported, which supports the thesis that evidence-based treatment of accidental hypothermia does not exist [17]. Extracorporeal rewarming was not undertaken in any of the facilities. Experience obtained in leading medical centres shows that noninvasive rewarming is both effective and safe and that indications for invasive methods are restricted to only a few cases [5,8,14-17]. It should be emphasized however that extracorporeal rewarming may be crucial for the patient’s survival in severe hypothermia accompanied by cardiovascular instability [13,17-20]. Cardiopulmonary resuscitation was required at the initial stage of therapy in 20 out of 268 patients included in our study. Given the experience obtained at University Hospital in Vienna [16,20], it may be presumed that extracorporeal rewarming was potentially indicated in at least half of the group i.e. in about 4% of the patients who died.

The methods of extracorporeal blood rewarming are available in a limited number of specialized medical centres. In practice, they can be applied only in patients who developed hypothermia in the neighbourhood of cardiothoracic surgical centres. Even though patients could be transported from distant hospitals, this is not always feasible and requires a procedure which is complex in terms of organisation [21]. Otherwise, medical equipment may be needed for current surgical procedures and thus not be available for other purposes. All these factors contribute to the fact that extracorporeal rewarming is rarely an option for ER personnel. It may be expected that an information campaign and the development of management procedures including transport to specialized facilities will increase the survival rate in accidental hypothermia [13,19].

This is the first ever survey on accidental hypothermia victims in Poland. The survey confirmed our personal observations that the current methods of diagnosis and treatment of accidental hypothermia are probably far from perfect. Therefore, it might be reasonable to establish regional centres designated to providing expert help and organizational support, as well as to promote effective means of treatment especially in severe stages of accidental hypothermia. Recently, based on preliminary results of a current study, the Severe Hypothermia Treating Centre has been established in Cracow. It is the first and so far the only specialized unit in Poland dedicated to the extracorporeal treatment of accidental hypothermia [22]. The centre cooperates with the International Hypothermia Registry [23] We hope that based on the Centre’s experience and organization, other provinces in Poland will make the effort to create similar facilities.

### Limitations

Considering the character of the study and the research method applied, a certain margin of data error is acceptable here. In addition, we obtained data from only about 20% of the ERs which provide healthcare to less than 15% of the population in Poland. Therefore, the presented data and analysis should be seen as a rough guide. The study was limited to the treatment phase within the ER. As a result, we do not know the follow-up history of the patients once they have been transferred to other departments. Consequently, it is impossible to give an opinion on overall hypothermia-related hospital morbidity. We did not find any studies with the same design which might be used as a reference. We used analyses of national databases and data collected in single clinics or groups of medical centres for this purpose. Considering that core temperature was taken in only 1/3 of patients, data on hypothermia classification cannot be completely reliable.

## Conclusions

The actual incidence of accidental hypothermia in Polish emergency departments may exceed the official data by up to four times. The diagnosis of hypothermia is based on the peripheral temperature measurement and clinical examination in two thirds of the cases, while the core temperature is taken in only one third of the patients. Several rewarming options are applied with the use of a variety of equipment, which supports the thesis that evidence-based treatment of accidental hypothermia does not exist. The treatment of hypothermic patients is rarely conducted in intensive care units, and extracorporeal rewarming techniques are not used. It may be expected that personnel education and the development of management procedures, including transport to specialized facilities, will brighten the prognosis and increase the survival rate in accidental hypothermia.

## References

[1] Baumgartner EA, Belson M, Rubin C, Patel M (2008). Hypothermia and other cold-related morbidity emergency department visits: United States, 1995–2004. Wilderness Environ Med.

[2] Bierens JJ, Uitslager R, Swenne-Van Ingen MM, Van Stiphout WA (1995). Accidental hypothermia: incidence, risk factors and clinical course of patients admitted to hospital. Eur J Emerg Med.

[3] Herity B, Daly L, Bourke GJ, Horgan JM (1991). Hypothermia and mortality and morbidity. an epidemiological analysis. J Epidemiol Community Health.

[4] Vassal T, Benoit-Gonin B, Carrat F, Guidet B (2001). Severe accidental hypothermia treated in an ICU. Chest.

[5] Brändström H, Johansson G, Giesbrecht GG, Ängquist KA, Haney MF (2014). Accidental cold-related injury leading to hospitalization in northern Sweden: an eight-year retrospective analysis. Scand J Trauma Resusc Emerg Med.

[6] Taylor NA, Griffiths RF, Cotter JD (1994). Epidemiology of hypothermia: fatalities and hospitalisations in New Zealand. NZ J Med.

[7] Hislop LJ, Wyatt JP, McNueghton GW, Ireland IJ (1995). Urban hypothermia in the West of Scotland. BMJ.

[8] Danzl DF, Pozos RS, Auerbach PS, Glazer S (1987). Multicenter hypothermia survey. Ann Emerg Med.

[9] Soar J, Perkins GD, Abbas G, Alfonzo A, Barelli A, Bierens JJLM (2010). European resuscitation council guidelines for resuscitation 2010 section 8. cardiac arrest in special circumstances: Electrolyte abnormalities, poisoning, drowning, accidental hypothermia, hyperthermia, asthma, anaphylaxis, cardiac surgery, trauma, pregnancy, electrocution. Resuscitation.

[10] Durrer B, Brugger H, Syme D (2003). The medical on-site treatment of hypothermia ICAR-MEDCOM recommendation. High Alt Med Biol.

[11] Strapazzon G, Procter E, Paal P, Brugger H (2014). Pre-hospital core temperature measurement in accidental and therapeutic hypothermia. High Alt Med Biol.

[12] Walpoth BH, Galdikas J, Leupi F, Muehlemann W, Schlaepfer P, Althaus U (1994). Assessment of hypothermia with a new “tympanic” thermometer. J Clin Monit.

[13] Brown DJ, Brugger H, Boyd J (2012). Accidental hypothermia. N Engl J Med.

[14] Megarbane B, Axler O, Chary I, Pompier R (2000). Hypothermia with indoor occurrence is associated with a worse outcome. Intensive Care Med.

[15] Ledingham IM, Mone JG (1980). Treatment of accidental hypothermia: a prospective clinical study. BMJ..

[16] Roeggla M, Holzer M, Roeggla G, Frosard M (2001). Prognosis of accidental hypothermia in the urban setting. J Intensive Care Med.

[17] van der Ploeg GJ, Goslings JC, Walpoth BH, Bierens JJ (2010). Accidental hypothermia: rewarming treatments, complications and outcomes from one university medical centre. Resuscitation.

[18] Ruttmann E, Weissenbacher A, Ulmer H, Müller L (2007). Prolonged extracorporeal membrane oxygenation-assisted support provides improved survival in hypothermic patients with cardiocirculatory arrest. J Thorac Cardiovasc Surg.

[19] Paal P, Brown D (2014). Cardiac arrest from accidental hypothermia, a rare condition with potentially excellent neurological outcome, if you treat it right. Resuscitation.

[20] Schober A, Sterz F, Handler C, Kürkciyan I, Laggner A, Röggla M (2014). Cardiac arrest due to accidental hypothermia-a 20 year review of a rare condition in an urban area. Resuscitation.

[21] Holmström P, Boyd J, Sorsa M, Kuisma M (2005). A case of hypothermic cardiac arrest treated with an external chest compression device (LUCAS) during transport to re-warming. Resuscitation.

[22] Darocha T, Kosiński S, Jarosz A, Gałązkowski R, Sadowski J, Drwiła R. Severe accidental hypothermia center. Eur J Emerg Med. 2014. doi:10.1097/MEJ.0000000000000213 10.1097/MEJ.0000000000000213 25304125

[23] International Hypothermia Registry. https://www.hypothermia-registry.org.

